# Comparison of organic and inorganic hole transport layers in double perovskite material-based solar cell

**DOI:** 10.3762/bjnano.16.11

**Published:** 2025-02-06

**Authors:** Deepika K, Arjun Singh

**Affiliations:** 1 Department of Applied Sciences, The Northcap University, Gurugram, India

**Keywords:** double perovskite solar cell (DPSC), electron transport layer (ETL), hole transport layer (HTL), SCAPS-1D, simulation

## Abstract

Perovskite solar cells (PSCs) are in the focus of the photovoltaic industry. Lead-free double perovskite solar cells (DPSCs) have become an essential alternative of lead-based PSCs as a promising photovoltaic material. The double perovskite layer is a remarkable choice as active layer because of intrinsic carrier stability, low exciton binding energy, and low toxicity. Herein, the optimization of a planar DPSC with a multifunctional double perovskite absorber layer, that is, La_2_NiMnO_6_ (LNMO), is studied with the organic and inorganic hole transport layers (HTLs) Cu_2_O and PEDOT:PSS. Our study yields a significant improvement in the power conversion efficiency (PCE) of perovskite solar cells with two types of HTLs. The optimized devices achieved a maximum PCE of 27.84% and 27.38% for Cu_2_O and PEDOT:PSS, respectively, with corresponding open-circuit voltages of 1.27 and 1.22 V, short-circuit current densities of 28.60 and 28.91 mA/cm^2^, and fill factors of 76.31% and 77.15%, respectively. These results highlight the potential of these HTLs for enhanced device performance.

## Introduction

The rapid growth of the world population has increased the global need for energy, which has become undoubtedly quite strong. To date, the energy requirements have been mostly fulfilled by conventional sources of energy, which include a major portion of fossil fuels. But nowadays, environmentally clean energy sources are moving forward. During the past few decades, various clean energy forms have been introduced, which include wind energy and hydropower, as well as geothermal, tidal, and solar energy. Renewable energy sources are unlimited and can be constantly replenished. In the coming years, renewable energy sources will contribute to decarbonizing energy systems. Solar energy safeguards both human health and a healthy environment [1]. Akmam and Karapinar [2] fabricated a dye-sensitized solar cell (DSSC) with selenium@activated carbon (Se@AC) composites as an alternative to the Pt counter electrode (CE) via chemical activation. The fabricated DSSC showed a power conversion efficiency (PCE) of 5.67%, an open-circuit voltage (*V*_OC_) of 0.648 V, a short-circuit current density (*J*_SC_) of 13.26 mA/cm^2^, and a fill factor (FF) of 66%. The PCE is close to that of the Pt-based counter electrode (PCE = 6.86%). Akman [3] used hydrothermal methods to synthesize the photoanodes with different doping sources to further improve the stability of DSSCs. For 1.0 mol % Mn doping and an Eu compact layer, an efficiency of 4.20% was obtained.

Currently, perovskite solar cells (PSCs) are attracting the attention of research communities worldwide because of their outstanding and unique properties. PSCs possess desirable characteristics such as cost-effectiveness, extended carrier diffusion lengths, and adjustable direct bandgaps. Also, there are well-established fabrication techniques that have positioned PSCs as a solution-processable photovoltaic technology [4]. Over the past few years, a significant improvement in the PCE of the PSCs was reported, from 3.8% in 2009 to 26.1% in 2023 [5–6]. PSCs consist of an absorber layer sandwiched between charge transport layers (CTLs), that is, the hole transport layer (HTL) and the electron transport layer (ETL). Light generates excitons, which further dissociate into electrons and holes. The electrons and holes are transported to ETL and HTL, respectively, without recombining [7]. Ozturk et al. [8] addressed the role of a passivation agent at grain boundaries and the surface of perovskite films, namely, quinary kesterite nanocrystals Cu_2_NiSn(S,Se)_4_ (CNTSSe) obtained through a facile hot-casting method. Through passivation, efficiencies of 20.8% for Cs_0.05_(FA_0.90_MA_0.10_)_0.95_Pb(I_0.90_Br_0.10_)_3_, 18.9% for MAPbI_3_, and 18.7% for FAPbI_3_ perovskite layers were observed under ambient conditions and illumination for over 900 h. Mohammed et al. [9] optimized triple-cation PSCs that maintained 83% of the efficiency after 1600 h under ambient conditions with humidity levels of 35–40%. Here, 3,4-dihydroxyphenethylamine hydrochloride (3,4-DpACl) was used as an additive during perovskite fabrication.

Despite significant research efforts, there are stability issues when working under critical environmental conditions, which is an essential issue for practical applications in the future. The structure of PSCs is ABX_3_, where A and B are the cationic sites and X is the anionic site. In double perovskite solar cells (DPSCs), the unit cell is twice that of the perovskite, that is, A_2_BB′O_6_. It has two cations at the sites B and B′ with corner-sharing BO_6_ and B′O_6_ units featuring a rock salt-like arrangement [10–11]. The commercialization of PSCs is impeded because of toxicity and long-term instability. DPSCs turned out to be better than PSCs because of better tunability, higher environmental stability, and higher efficiency. In DPSCs, the double perovskite layer is sandwiched between the CTLs. In 2021, Kumar et al., reported a PCE of 9.68% for a La_2_NiMnO_6_ (LNMO)-based device structure after the bandgap had been optimized using the SCAPS-1D software [12]. In 2022, Porwal et al. reported a PCE of 23.64% with Cs_2_SnI_6_ as double perovskite layer calculated via SCAPS-1D [13]. In 2023, Alla et al. simulated, using SCAPS-1D, a Cs_2_CuSbX_6_-based DPSCs with an efficiency exceeding 29% [14].

In 2023, Singh et al. optimized a lead-free DPSC with La_2_NiMnO_6_ as absorber layer, Cu_2_O as HTL, and ZnOS as an ETL. They obtained an efficiency of 25.44% with *V*_OC_ = 1.1027 V, *J*_SC_ = 27.89 mA/cm^2^, and FF = 82.69%, suggesting the suitability of La_2_NiMnO_6_. In 2024, Singh et al. proposed a planar DPSC with La_2_NiMnO_6_ as absorber layer, Cu_2_O as HTL, and WS_2_ as ETL, and several parameters of the absorber layer including thickness, defect density, series and shunt resistance, interfacial defect density, and various metal electrodes were studied. An efficiency of 18.89% with *V*_OC_ = 0.7919 V, *J*_SC_ = 27.89 mA/cm^2^, and FF = 85.52% was reported for the device structure FTO/WS_2_/La_2_NiMnO_6_/Cu_2_O/Au [15–16]. In 2023, the highest optimized efficiency of 24.08% was reported for the device configuration FTO/WS_2_/LNMO/Cu_2_O/Au, representing La_2_NiMnO_6_ as an eco-friendly and non-toxic oxide material usable for further applications [17].

In literature, DPSCs with inorganic Cu_2_O have been studied, but in this manuscript we also consider organic materials. The optimized PSC device displays a higher efficiency of 27.84% with Cu_2_O and 27.38% with PEDOT:PSS for the planar n-i-p FTO/WS_2_/LNMO/HTL/Au device structure. However, highly efficient organic HTLs have a few disadvantages over inorganic HTLs, including multistep synthesis requiring additional doping, leading to device instability [18]. In this manuscript, La_2_NiMnO_6_ (LNMO) is used as a double perovskite light absorbing layer for the device structure FTO/WS_2_/LNMO/HTL/Au, where WS_2_ is used as ETL. We optimize the La_2_NiMnO_6_ double perovskite with respect to organic (PEDOT:PSS) and inorganic (Cu_2_O) HTLs. This work mainly explains the impact of HTLs on the double perovskite material because, until now, the efficiency is low in this type of solar cell. The results highlight the potential of these HTLs for enhanced device performance in DPSCs. Also, the optimized parameters from these studies indicate pathways for experimental work regarding better performance.

## Simulation Methodology and Device Structure

**Figure 1:**
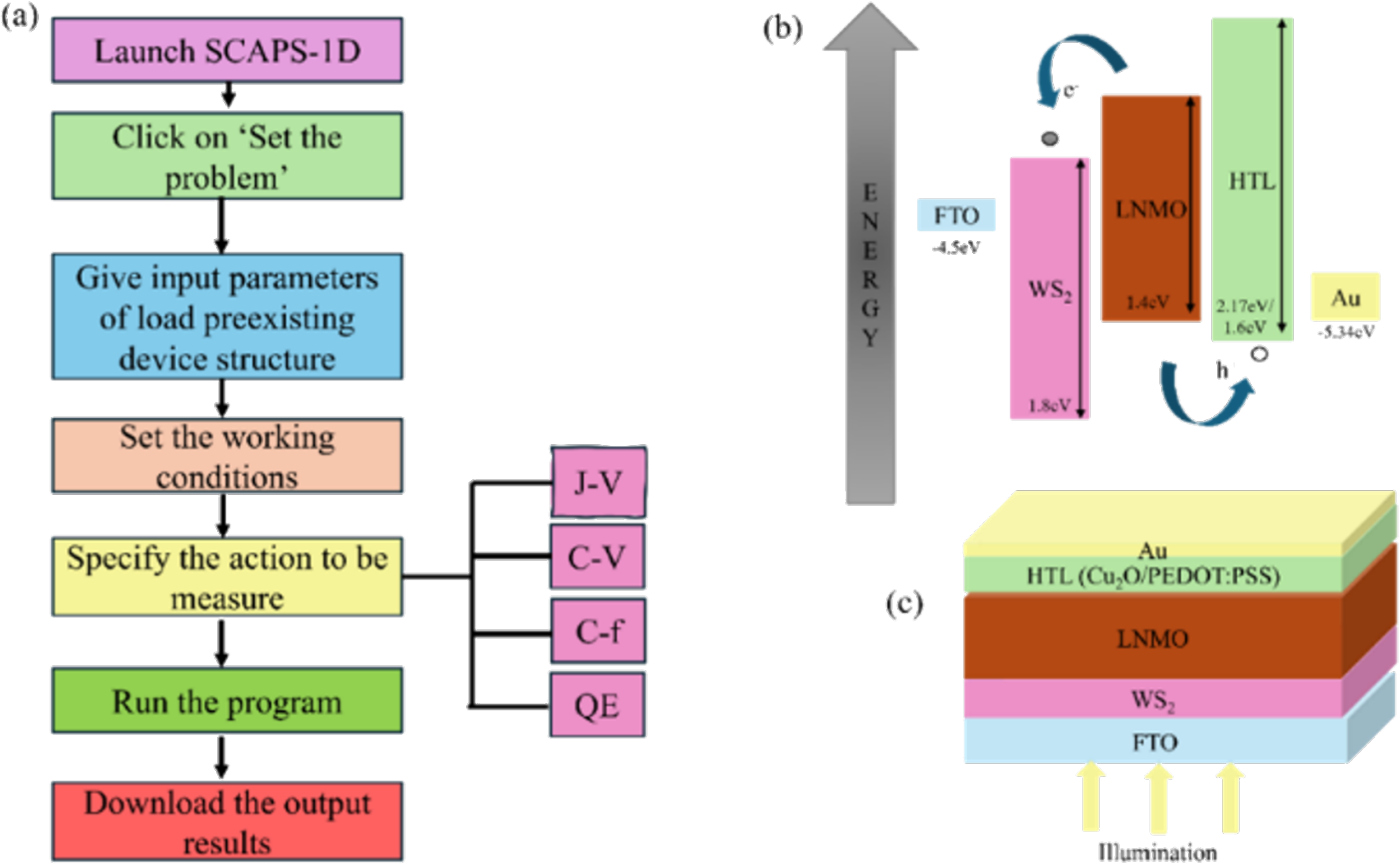
(a) Methodology of SCAPS-1D program, (b) energy band diagram of the planar n-i-p DPSC, and (c) planar n-i-p device structure of DPSC.

SCAPS-1D (a solar cell capacitance simulator) is an application program in one-dimensional C code developed at the Electronics and Informative Systems Department of Gent University, Belgium. It facilitates the modeling of graded device structures up to seven layers and the computing of device parameters such as bandgap energy, efficiency, and *J*–*V* characteristics [19]. It helps in understanding the various functions of the device in more detail while indicating the aspects that have the highest impact on the device performance. SCAPS-1D uses some basic steps to obtain the device’s output characteristics. The SCAPS-1D program steps and the device structure are shown in Figure 1.

The device behavior can be studied and solved using SACPS-1D by solving 1D Poisson and continuity equations. The Poisson equation is as follows [20]:


[1] $$\frac{{\text{d}}^{2}\phi \left(x\right)}{\text{d}x^{2}}=\frac{e}{\varepsilon_{0}\varepsilon_{\text{r}}}\left(p\left(x\right)-n\left(x\right)+N_{\text{D}}-N_{\text{A}}+\rho_{\text{p}}-\rho_{\text{n}}\right),$$

where *e* is the electronic charge, ϕ is the electric potential, ε_0_ is the vacuum permittivity, ε_r_ is the relative permittivity, *p*(*x*) and *n*(*x*) are, respectively, hole and electron position dependence, *N*_D_ is the shallow donor density, *N*_A_ is the acceptor donor density, and ρ_p_ and ρ_n_ are, respectively, the free density distributions of holes and electrons. The Poisson equation explains the change in electric field with respect to charge densities. The continuity equations are [21]:


[2] $$\frac{\text{d}J_{\text{n}}}{\text{d}x}=G-R,$$

[3] $$\frac{\text{d}J_{\text{p}}}{\text{d}x}=G-R,$$

where *J*_n_ and *J*_p_ are electron and hole density, respectively, *G* is the generation rate of free e^−^ and h^+^, and *R* is the recombination rate of e^−^ and h^+^ per unit volume.

The continuity equations consider the generation, recombination, and movement of carriers [22]. Electrical performance can be derived using the above equations. The differential form of both equations represents microscopic material behavior. The device simulation is performed at an air mass of AM1.5G at 300 K under illumination of 1000 W/m^2^. The absorber layer is optimized concerning different hole transport layers with the help of SCAPS-1D. The materials and the proposed parameters, taken from different publications, for this study are given in Table 1.

**Table 1:** Parameters for the optoelectronic simulation of planar device.

Parameter	FTO	WS_2_	LNMO	Cu_2_O	PEDOT:PSS

thickness (nm)	300	50	350	250	100
bandgap (eV)	3.4	1.8	1.4	2.17	1.6
electron affinity (eV)	4.5	3.95	3.52	3.2	3.55
relative permittivity	9.1	13.6	3.5	7.11	2.58
effective density of states at CB (cm^−3^)	1.1 × 10^19^	2.2 × 10^16^	1 × 10^18^	1.1 × 10^19^	2.1 × 10^21^
effective density of states at VB (cm^−3^)	1.1 × 10^19^	2.2 × 10^17^	1 × 10^18^	2.02 × 10^17^	2.0 × 10^21^
electron thermal velocity (cm/s)	1 × 10^7^	1 × 10^7^	1 × 10^7^	1 × 10^7^	1 × 10^7^
hole thermal velocity (cm/s)	1 × 10^7^	1 × 10^7^	1 × 10^7^	1 × 10^7^	1 × 10^7^
electron mobility (cm^2^/Vs)	20	100	22	200	1
hole mobility (cm^2^/Vs)	10	100	22	80	20
density of n-type doping *N*_D_ (cm^−3^)	1.1 × 10^19^	1 × 10^18^	0	0	0
density of p-type doping *N*_A_ (cm^−3^)	0	0	7 × 10^16^	1 × 10^18^	3 × 10^20^
density of defects *N*_t_ (cm^−3^)	donor – 1 × 10^14^	1 × 10^15^	1 × 10^14^	1 × 10^14^	1 × 10^14^
reference	[23]	[16]	[16]	[16]	[24]

## Experimental Verification

In 2024, a lead-free DPSC was both designed and fabricated. The included LNMO material was synthesized using the sol–gel method. The experimental and simulated *J*–*V* curves showed PCEs of 4.5 % and 10%, respectively. For the simulation, TiO_2_ was used as ETL, and NiO was used as HTL, with La_2_NiMnO_6_ as absorber [25]. The DPSC showed promising characteristics. Applications of double perovskite compounds include fuel cells, UV sensors, electrochemical sensors, indoor photovoltaics, and light-emitting diodes [26]. Double perovskite LNMO nanoparticles and nanorods were synthesized via a hydrothermal process, and it was found that they had a larger saturation magnetization [27]. Simulation results yielded PCEs of over 26%, but there are still challenges associated with the application of DPSCs [28].

## Results and Discussion

In this simulation study, Cu_2_O and PEDOT:PSS are the two HTLs that are analyzed concerning the double perovskite material LNMO. The HTL needs better conductivity, better electron blocking, and more hole mobility for better carrier transportation at the perovskite/HTL interface. It is hydrophobic with a wider bandgap and does not easily deteriorate. Inorganic HTLs proved to perform better. Some examples of inorganic HTLs are CuI, Cu_2_O, and CuSCN. Organic HTLs consist of polymers or complex molecules, which affect the photovoltaic properties of the device in terms of light absorption and carrier mobility. Some examples of organic materials are PEDOT:PSS, P3HT, Spiro-OMeTAD, and PTAA. Our simulations were performed with Cu_2_O and PEDOT:PSS HTLs [24,29–30].

### Effect of the absorber layer thickness

The absorber layer in a PSC is sandwiched between the CTLs such that upon illumination the formed electrons and holes get captured by the corresponding CTL [21]. The thickness of the absorber layer plays a crucial role regarding the device performance. In this simulation, the thickness of the absorber layer was varied from 250 to 650 nm for different HTLs, and the resulting PCE was calculated. Along with the absorber layer, the HTL is also an important part of a PSC. It blocks electrons, and only holes are captured to make carrier transportation feasible across the perovskite/HTL interface. Here, the impact of both inorganic Cu_2_O and organic PEDOT:PSS as HTLs is studied.

**Figure 2:**
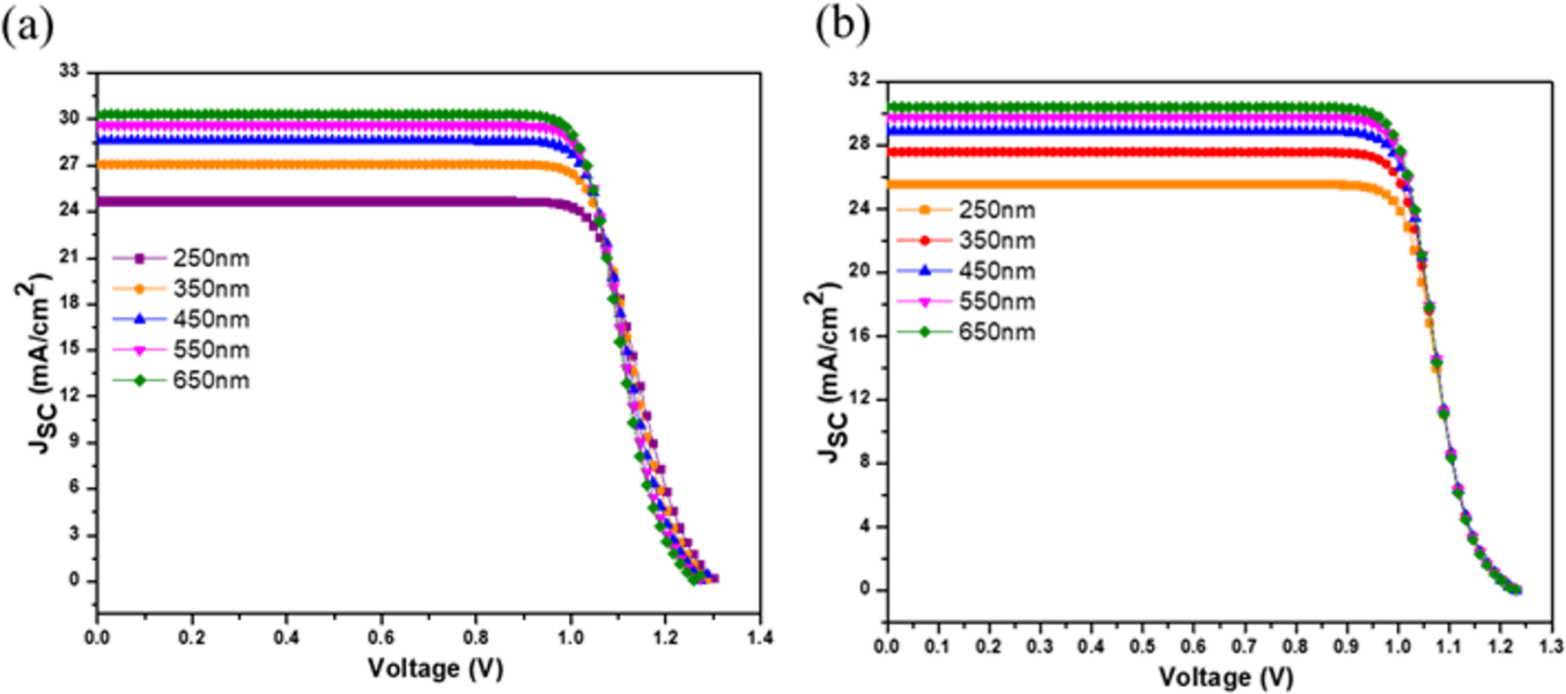
Optimization of the absorber layer with (a) Cu_2_O and (b) PEDOT:PSS.

The simulation of the FTO/WS_2_/LNMO/Cu_2_O/Au device with increasing thickness of the absorber layer from 250 to 650 nm yielded increases in *J*_SC_ from 24.66 to 30.31 mA/cm^2^ and in PCE from 24.46% to 29.16% (Table 2). A slight drop in *V*_OC_ from 1.29 to 1.26 V with no change in the fill factor was observed as well. In the case of PEDOT:PSS, increases in *J*_SC_ from 25.55 to 30.42 mA/cm^2^ and PCE from 24.33% to 28.69% were observed with no variations in *V*_OC_ or FF (Figure 2). The saturation of the FF in the devices with both HTLs signifies the increase of the series resistance. The greater the thickness of the DPSC layer, the more light is absorbed, generating a larger number of excitons [31].

**Table 2:** Obtained device parameters at different absorber layer thicknesses with Cu_2_O and PEDOT:PSS as HTLs.

	Cu_2_O				PEDOT:PSS			
Thickness (nm)	*V*_OC_ (V)	*J*_SC_ (mA/cm^2^)	FF (%)	PCE (%)	*V*_OC_ (V)	*J*_SC_ (mA/cm^2^)	FF (%)	PCE (%)

250 nm	1.29	24.66	76.50	24.46	1.22	25.55	77.52	24.33
350 nm	1.28	27.07	76.35	26.55	1.22	27.59	77.30	26.20
450 nm	1.27	28.60	76.31	27.84	1.22	28.91	77.15	27.38
550 nm	1.26	29.62	76.27	28.64	1.22	29.80	77.03	28.16
650 nm	1.26	30.31	76.26	29.16	1.22	30.42	76.95	28.69

### Effect of the device temperature

**Figure 3:**
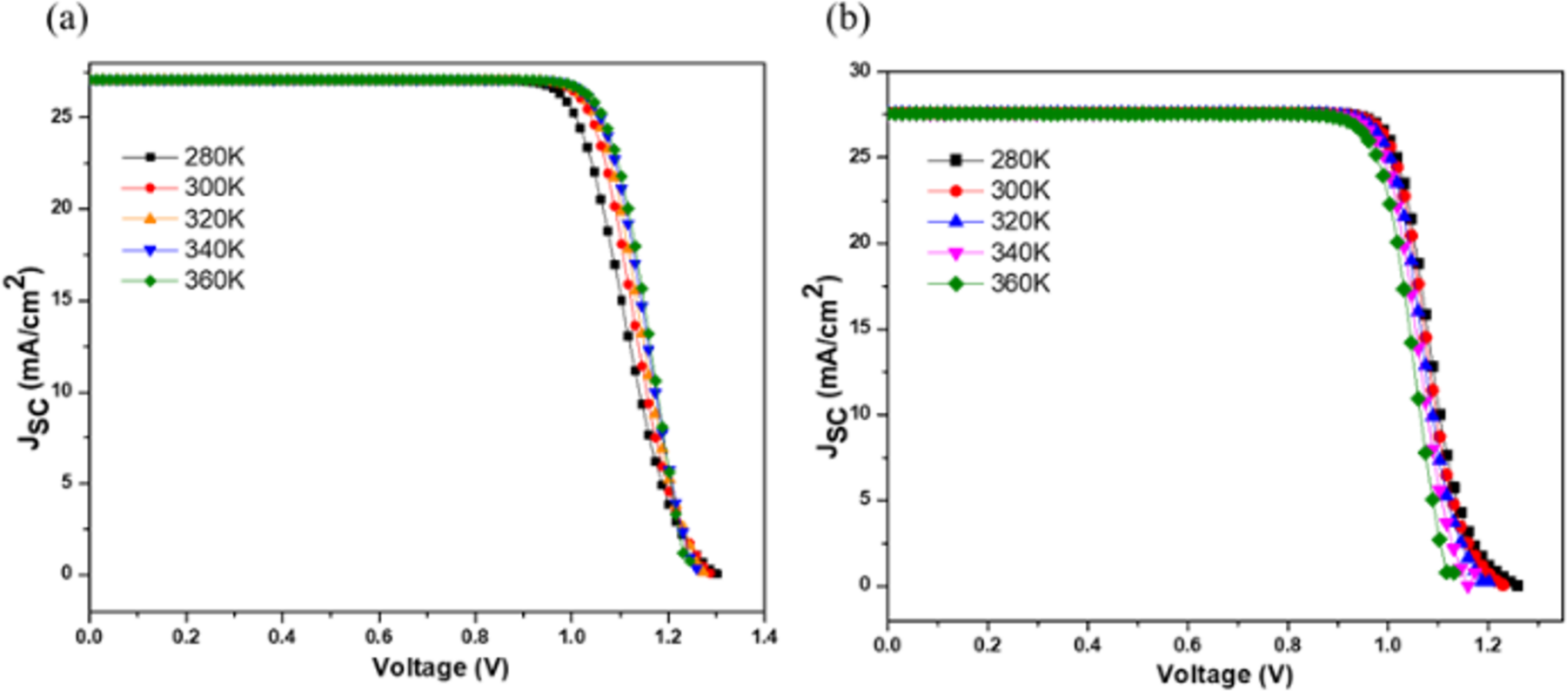
Optimization of device temperature with (a) Cu_2_O and (b) PEDOT:PSS.

The device temperature is the pivotal factor influencing the performance of PSCs. The standard temperature to be considered while simulating the device structure is 300 K [32]. To study the impact of the temperature on the electrical parameters of the proposed device setup, the temperature was varied from 280 to 360 K. With the increase in temperature above room temperature, the PCE decreased from 26.37% to 25.14% with PEDOT:PSS; but, surprisingly, an increase in PCE from 25.68% to 27.10% was observed in the case of Cu_2_O along with an increase in FF for both HTL materials (Table 3 and Figure 3). The increase of the PCE with Cu_2_O signifies a better absorption of light with minimum reflection and, hence, increased carrier transportation across the interface. Cu_2_O is inorganic, and the high temperature leads to improved hole mobility and better charge transport across the Cu_2_O/absorber layer interface. The increase in the device temperature decreases the efficiency of the PEDOT:PSS-based device. This may be due to the deterioration of the PEDOT:PSS/absorber layer interface at elevated temperatures, increasing trap-assisted recombination. Also, it is possible that misalignment of energy levels due to thermal effects can hinder efficient hole extraction, further increasing recombination losses.

**Table 3:** Obtained device parameters with Cu_2_O and PEDOT:PSS as HTLs at different temperatures.

	Cu_2_O				PEDOT:PSS			
Temperature (K)	*V*_OC_ (V)	*J*_SC_ (mA/cm^2^)	FF (%)	PCE (%)	*V*_OC_ (V)	*J*_SC_ (mA/cm^2^)	FF (%)	PCE (%)

280	1.29	27.07	73.07	25.68	1.25	27.60	75.93	26.37
300	1.28	27.07	76.35	26.55	1.22	27.59	77.30	26.20
320	1.26	27.07	78.06	26.84	1.19	27.58	78.61	25.92
340	1.25	27.08	79.52	27.02	1.16	27.57	79.92	25.57
360	1.23	27.08	80.77	27.10	1.12	27.56	81.14	25.14

### Effect of absorber layer defect density

**Figure 4:**
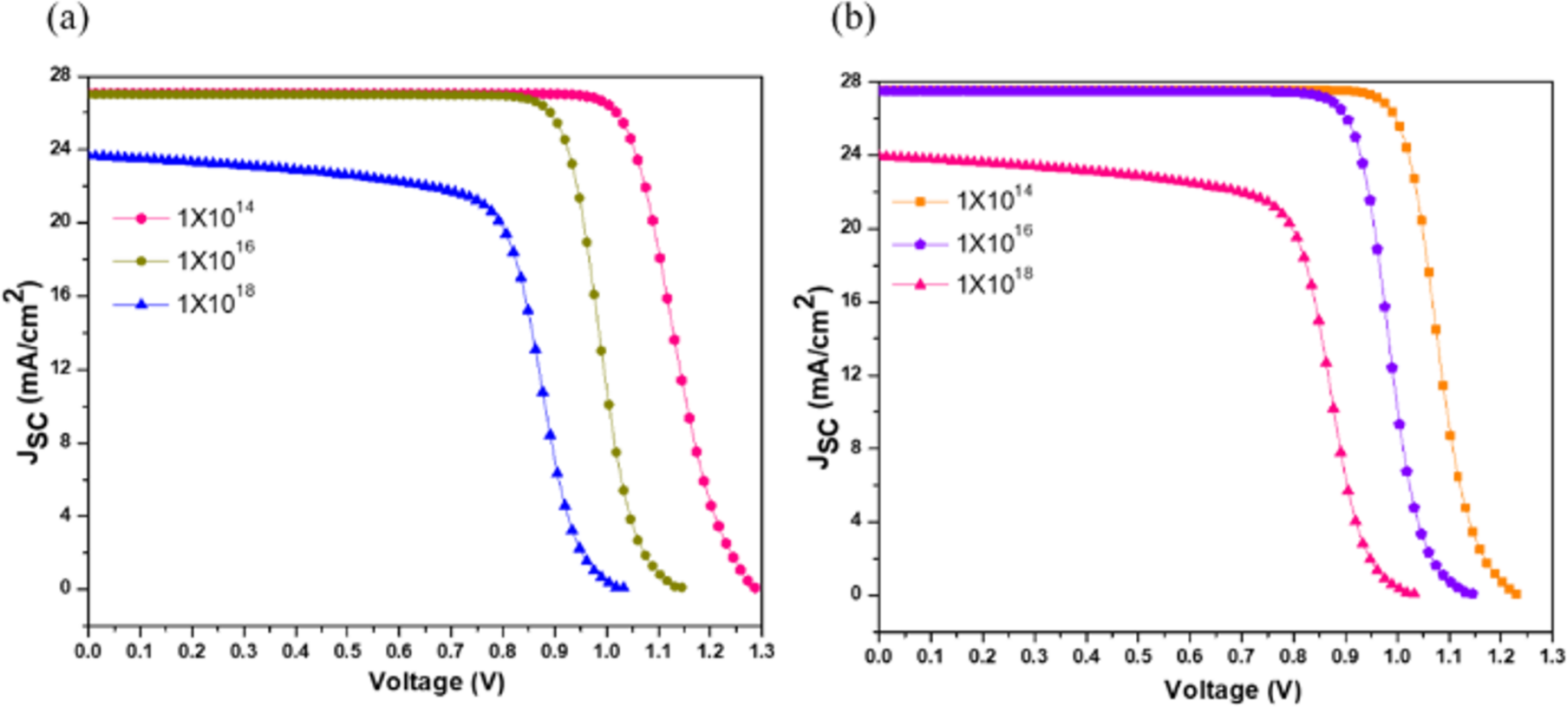
Optimization of total defect density of absorber layer with (a) Cu_2_O and (b) PEDOT:PSS.

Defects in the absorber layer hinder carrier transportation between the absorber layer and the CTLs, leading to a drop in the device performance [33–34]. Here, simulations were carried out for defect densities of 1 × 10^14^, 1 × 10^16^, and 1 × 10^18^ cm^−3^ for both HTLs (Table 4 and Figure 4).

**Table 4:** Obtained device parameters for various defect densities of the absorber layer with Cu_2_O and PEDOT:PSS as HTLs.

	Cu_2_O				PEDOT:PSS			
Total defect density (*N*_t_) (cm^−3^)	*V*_OC_ (V)	*J*_SC_ (mA/cm^2^)	FF (%)	PCE (%)	*V*_OC_ (V)	*J*_SC_ (mA/cm^2^)	FF (%)	PCE (%)

1 × 10^14^	1.28	27.06	76.36	26.54	1.22	27.59	77.30	26.20
1 × 10^16^	1.13	27.01	75.34	23.19	1.14	27.54	75.29	23.65
1 × 10^18^	1.02	23.66	66.06	16.05	1.02	23.96	65.81	16.21

Table 4 shows that there is a decrement in all output parameters of the device with *N*_t_; therefore, 1 × 10^14^ cm^−3^ was chosen as the optimum value. The increase in the defects reduces the absorber film’s overall quality because trap sites are generated. Both Cu_2_O and PEDOT:PSS show different results at 1 × 10^18^ cm^−3^; the PCE is 16.05% for Cu_2_O and 16.21% for PEDOT:PSS, implying a higher recombination rate in the case of the inorganic HTL.

### Optimized results

**Figure 5:**
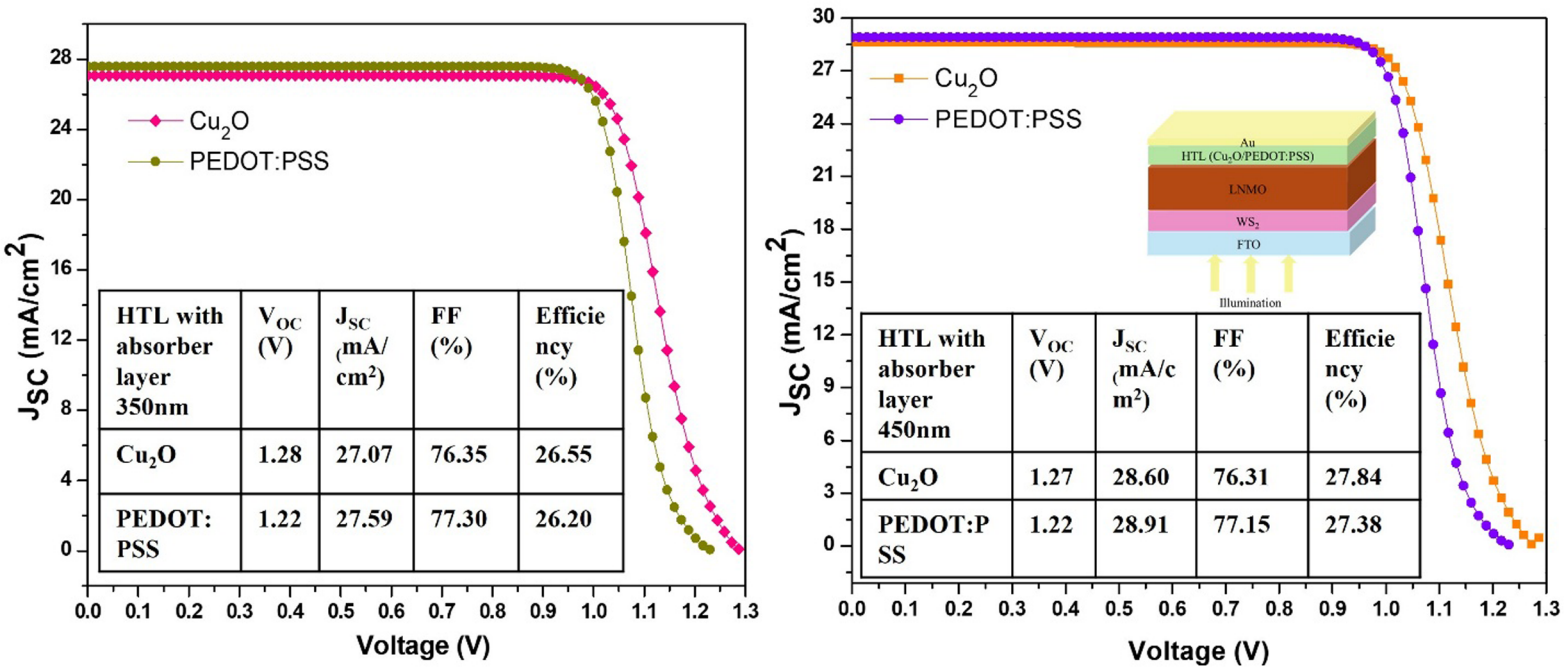
(a) Literature-based device results and (b) optimized results with Cu_2_O and PEDOT:PSS as HTL materials.

The work presented in this publication starts with a literature-based device. The simulation parameters of each layer were taken directly from literature as shown in Table 1. After that, the simulation parameters were optimized. Table 5 and Figure 5 compare the optimized results with the literature-based device. The maximum efficiency reported was 27.84% after optimization for Cu_2_O.

**Table 5:** Literature-based device and optimized parameters of the planar device FTO/WS_2_/LNMO/HTL/Au with both inorganic and organic HTL materials.

Output parameters	Literature-based device: Cu_2_O	Literature-based device: PEDOT:PSS	Optimized device: Cu_2_O	Optimized device: PEDOT:PSS

*V*_OC_ (V)	1.28	1.22	1.27	1.22
*J*_SC_ (mA/cm^2^)	27.07	27.59	28.60	28.91
FF (%)	76.35	77.30	76.31	77.15
PCE (%)	26.55	26.20	27.84	27.38

The SCAPS-1D model is a useful tool to simulate how perovskite solar cells work. However, it has some drawbacks we need to keep in mind when we look at the results. One big issue is that it assumes perfect material properties, such as uniformity in thickness, defect density, and material interfaces, which do not represent real-world conditions. Further, complicated interfacial effects between the active layer and the HTLs, such as chemical interactions, degradation, or the existence of intermediary defect states that may affect carrier recombination and transport, are not fully taken into account by the model. Since their physical and chemical interactions with the perovskite layer can have a big impact on device performance, these factors are especially important when comparing various HTL materials. Future work should aim to incorporate these considerations through experimental validation and more sophisticated simulation approaches, such as multidimensional modelling or coupling SCAPS-1D with experimental datasets. These efforts would enhance the reliability of theoretical predictions and their applicability to practical device development.

## Conclusion

Solar energy is one of the most promising renewable energy sources. It is one of those energy sources creating no harmful emissions. PSCs are a new generation in the photovoltaic industry. Here, a La_2_NiMnO_6_ (LNMO)-based DPSC is simulated with optimized device temperature, absorber layer thickness, and defect density (*N*_t_) parameters. The incorporation of Cu_2_O and PEDOT:PSS significantly enhances device performance with PCEs of 27.84% and 27.38%, respectively. These improvements are supported by key metrics, including *V*_OC_ values of 1.27 and 1.22 V, *J*_SC_ values of 28.60 and 28.91 mA/cm^2^, and FFs of 76.31% and 77.15%, respectively. These findings underscore the potential of these HTLs to drive advancements in perovskite solar cell technology. The optimum result is obtained for the planar n-i-p FTO/WS_2_/LNMO/HTL/Au device structure at 300 K, with an absorber layer thickness of 450 nm and *N*_t_ = 1 × 10^14^ cm^−3^. It has been determined that with the increase of the width of the perovskite layer, the functionality of the photovoltaic cell is increased.

## Data Availability

Data generated and analyzed during this study is available from the corresponding author upon reasonable request.

## References

[1] Ritchie H, Roser M, Rosado P (2020). Renewable Energy. Our World in Data.

[2] Akman E, Karapinar H S (2022). Sol Energy.

[3] Akman E (2020). J Mol Liq.

[4] Kung P-K, Li M-H, Lin P-Y, Jhang J-Y, Pantaler M, Lupascu D C, Grancini G, Chen P (2020). Sol RRL.

[5] Cao F, Bian L, Li L (2024). Energy Mater Dev.

[6] Liang Z, Zhang Y, Xu H, Chen W, Liu B, Zhang J, Zhang H, Wang Z, Kang D-H, Zeng J (2023). Nature.

[7] Gonzalez-Pedro V, Juarez-Perez E J, Arsyad W-S, Barea E M, Fabregat-Santiago F, Mora-Sero I, Bisquert J (2014). Nano Lett.

[8] Ozturk T, Sarilmaz A, Akin S, Dursun H, Ozel F, Akman E (2022). Sol RRL.

[9] Mohammed M K A, Abualsayed M I, Alshehri A M, Kumar A, Dehghanipour M, Sh Alnayli R, Aftab S, Akman E (2024). ACS Appl Energy Mater.

[10] Meulenberg W A, Ivanova M E, Serra J M, Roitsch S, Basile A, Nunes S P (2011). Proton-conducting ceramic membranes for solid oxide fuel cells and hydrogen (H2) processing. Advanced Membrane Science and Technology for Sustainable Energy and Environmental Applications.

[11] Rahim W, Cheng A, Lyu C, Shi T, Wang Z, Scanlon D O, Palgrave R G (2020). Chem Mater.

[12] Kumar M, Raj A, Kumar A, Anshul A (2021). Mater Today Commun.

[13] Porwal S, Paul M, Dixit H, Mishra S, Singh T (2022). Adv Theory Simul.

[14] Alla M, Bimli S, Manjunath V, Choudhary E, Sharma S, Wakale G R, Miglani A, Rouchdi M, Fares B (2023). Mater Today Commun.

[15] Singh A, Srivastava V, Agarwal S, Lohia P, Dwivedi D K, Umar A, Ibrahim A A, Akbar S, Baskoutas S, Dakua P K (2024). J Opt (Bristol, U K).

[16] Singh A, Srivastava V, Singh S, Sadanand, Rai S, Lohia P, Dwivedi D K, Agarwal S, Ouladsmane M, Hossain M K (2024). J Opt (Bristol, U K).

[17] Yadav N, Khare A (2023). Phys Scr.

[18] Li S, Cao Y-L, Li W-H, Bo Z-S (2021). Rare Met.

[19] Hussain S S, Riaz S, Nowsherwan G A, Jahangir K, Raza A, Iqbal M J, Sadiq I, Hussain S M, Naseem S (2021). J Renewable Energy.

[20] Deepika, Singh A, Verma U K, Ameen S (2024). J Phys Chem Solids.

[21] Hossain M K, Toki G F I, Kuddus A, Rubel M H K, Hossain M M, Bencherif H, Rahman M F, Islam M R, Mushtaq M (2023). Sci Rep.

[22] Biswas S K, Sumon M S, Sarker K, Orthe M F, Ahmed M M (2023). Adv Mater Sci Eng.

[23] Kumar A, Singh S (2020). Mod Phys Lett B.

[24] Mondal B K, Mostaque S K, Rashid M A, Kuddus A, Shirai H, Hossain J (2021). Superlattices Microstruct.

[25] Sangavi T, Vasanth S, Viswanathan C, Ponpandian N (2024). Chem Eng J.

[26] Obada D O, Akinpelu S B, Abolade S A, Okafor E, Ukpong A M, Kumar R S, Akande A (2024). Crystals.

[27] Yi K, Tang Q, Wu Z, Zhu X (2022). Nanomaterials.

[28] Muscarella L A, Hutter E M (2022). ACS Energy Lett.

[29] Deepika, Singh A, Verma U K, Tonk A (2023). Phys Status Solidi A.

[30] Pitchaiya S, Natarajan M, Santhanam A, Asokan V, Yuvapragasam A, Madurai Ramakrishnan V, Palanisamy S E, Sundaram S, Velauthapillai D (2020). Arabian J Chem.

[31] Padelkar S S, Vikram, Jasieniak J J, Simonov A N, Alam A (2024). Phys Rev Appl.

[32] Mesquita I, Andrade L, Mendes A (2019). ChemSusChem.

[33] Ouslimane T, Et-taya L, Elmaimouni L, Benami A (2021). Heliyon.

[34] Alkhammash H I, Mottakin M, Hossen M M, Akhtaruzzaman M, Rashid M J (2023). Semicond Sci Technol.

